# The Correlation Between Parkinson’s Disease and Rapid Eye Movement Sleep Behavior Disorder: A Systematic Review

**DOI:** 10.7759/cureus.17026

**Published:** 2021-08-09

**Authors:** Niki Shrestha, Rose Anne M Abe, Anum Masroor, Arseni Khorochkov, Jose Prieto, Karan B Singh, Maduka C Nnadozie, Muhammad Abdal, Lubna Mohammed

**Affiliations:** 1 Internal Medicine, California Institute of Behavioral Neurosciences & Psychology, Fairfield, USA; 2 Research, California Institute of Behavioral Neurosciences & Psychology, Fairfield, USA; 3 Psychiatry, California Institute of Behavioral Neurosciences & Psychology, Fairfield, USA; 4 Psychiatry, Psychiatric Care Associates, Englewood, USA; 5 Medicine, Khyber Medical College, Peshawar, PAK; 6 Emergency Medicine, California Institute of Behavioral Neurosciences & Psychology, Fairfield, USA

**Keywords:** parkinson' s disease, neurodegenerative disorders, rapid eye movement sleep disorder, dream enactment, bradykinesia

## Abstract

Parkinson’s disease (PD) is a neurodegenerative disease caused due to the destruction of dopaminergic neurons and the deposition of α-synuclein proteins, known as Lewy bodies. Generally, the diagnosis of PD is centered around motor symptoms. However, the early recognition of non-motor symptoms such as autonomic dysfunction, sleep disturbances, and cognitive and psychiatric disturbances are gaining increased attention for the early diagnosis of PD. Rapid eye movement (REM) sleep behavior disorder or REM sleep behavior disorder (RBD) is described as parasomnia, which is a condition of loss of normal muscle atonia causing the person to act out vivid dreams and it has been seen to be associated with the misprocessing of intercellular α-synuclein leading to neurodegenerative diseases such as PD. This review’s objective is to highlight the significance of RBD as a prodromal premotor marker for the early detection of PD. We used PubMed as our primary database to search for articles on May 2, 2021, and a total of 1849 articles were found in our initial search using keywords and medical subject heading (MeSH) keywords. Thereafter, we removed the duplicates, applied the inclusion/exclusion criteria, and did a quality appraisal to include 10 articles in this study. We concluded that the recognition and diagnosis of RBD are of paramount importance to detect early PD, and further longitudinal studies and clinical trials are of utmost importance to understand their correlation; also, treatment trials are needed to prevent the phenoconversion of RBD into PD.

## Introduction and background

Parkinson’s disease (PD) is the second most prevalent neurodegenerative disease in the adult population of age greater than 65 years, with a prevalence rate of 0.4% [1]. James Parkinson was the first person to describe PD in his work known as ‘Essay on Shaking Palsy’ in 1817 [2, 3]. Abnormal proteinaceous spherical bodies known as Lewy bodies are formed, leading to the degeneration of dopaminergic nigrostriatal neurons. This correlates with the neuropathological brain changes in PD, which cause motor impairments such as bradykinesia, rigidity, postural instability, and resting tremor in patients [4, 5]. Although early researches on PD focused on motor symptoms, recent advances in studies have shed light on the non-motor symptoms of PD which include olfactory loss, constipation, mood disorders, pain, autonomic disturbances, and sleep dysfunctions [5, 6]. 

It has been found that the high prevalence of sleep problems in PD patients causes a significant reduction in the quality of life and even early institutionalization [3]. Excessive daytime sleepiness (EDS), difficulty in initiating and maintaining sleep, parasomnias, restless leg syndrome (RLS), periodic limb movements of sleep, and obstructive sleep apnea are the sleep problems seen in PD patients [5]. Recent studies have highlighted rapid eye movement (REM) sleep behavior disorder or REM sleep behavior disorder (RBD) as a premotor marker for neurodegenerative diseases as it has been seen to be associated with the misprocessing of intercellular α-synuclein [7].

The sleep-wake homeostasis and circadian rhythms mediated by the suprachiasmatic nucleus located in the anterior hypothalamus control the body’s sleep. Physiologically, the stages of sleep are non-rapid eye movement (NREM), subdivided into N1, N2, and N3, and REM sleep, which runs cyclically throughout the night [5]. The characteristics of REM sleep include rapid movement of the eyes, twitching of the muscles of the face and limbs, atonia, cortical activation, and vivid dreaming [8]. In 1986, RBD was first described as parasomnia, which is a condition of loss of normal muscle atonia causing the person to act out vivid dreams [9]. Although the cause is unclear, studies have suggested that the disruption of the protective mechanism mediated by the brainstem, pedunculopontine nucleus, and locus ceruleus leads to the loss of muscle atonia in RBD causing dream enactment [2]. It is usually associated with undesired and forceful violent movements such as kicking, punching, falling, or jumping out of the bed, and vocalizations as well as emotional outcries; every so often increasing the risk of physical trauma and affecting the patient and bed partner’s sleep [8]. 

Neurodegenerative diseases such as PD, multiple system atrophy (MSA), dementia with Lewy bodies (DLB) are associated with idiopathic RBD (iRBD). The other causes of RBD can be secondary to underlying neurological pathologies and medications [10]. The prevalence of RBD in the general population is approximately 4.9% and can vary anywhere from 20-72% in PD patients [1]. In patients with iRBD, the development of parkinsonian syndrome was found to be about 40-66% within 10 years and 20 years respectively [2]. In RBD patients, the akinetic/rigid-dominant subtype of PD is commonly seen and they tend to exhibit more severe clinical presentations [10]. It has been seen that males are more often affected than females. The other factors that escalate the development of PD in RBD patients are the increasing age of the patient and the chronicity of the disease [11]. The diagnosis of RBD requires the history and polysomnographic demonstration of dream enactment and the treatment usually includes patient counseling, a safe sleeping environment to decrease the risk of injuries (removing sharp objects, placing soft cushions, mattress, cotton pads on the floor), and medications such as melatonin and clonazepam. However, RBD does not respond to dopaminergic therapy used in PD [2, 3].

As it has been seen that the diagnosis of PD is centered towards motor symptoms, through this particular review we would like to emphasize the strong correlation between RBD and PD, and insight into RBD as a potential early premotor marker of PD as shown by the recent advances in studies [5]. 

## Review

Method

Search Strategy and Data Extraction

This was a qualitative systematic review conducted according to the Preferred Reporting Items for Systematic Reviews and Meta-Analysis (PRISMA) 2009 guidelines. We used PubMed, PubMed Central, and Medline as our primary databases to search for articles on May 2, 2021. We used keywords like 'Parkinson disease', 'Bradykinesia', 'REM sleep behavior disorder', and medical subject heading (MeSH) keywords like 'Parkinson disease/etiology', 'Parkinson disease/metabolism', 'Parkinson disease/physiopathology', 'REM sleep behavior disorder/etiology', 'REM sleep behavior disorder/metabolism', and 'REM sleep behavior disorder/physiopathology' to search for relevant studies. After applying the keywords and MeSH keywords in different combinations, we were able to render a total of 1849 articles in PubMed. The keywords and MeSH keywords used in this review are demonstrated in Table 1 and Table 2.

**Table 1:** Keywords and their search results REM: Rapid eye movement

KEYWORDS	DATABASE	Number of Results
Parkinson disease	PUBMED	3599
Rigidity	PUBMED	2402
Bradykinesia	PUBMED	158
REM sleep behavior disorder	PUBMED	146
Parkinson disease and REM sleep behavior disorder	PUBMED	133
Dream enacting behavior	PUBMED	22
Bradykinesia and REM sleep behavior disorder	PUBMED	6

**Table 2:** MeSH keywords and their search results MeSH: Medical subject headings; REM: Rapid eye movement

MeSH Keywords	Number of Results
"Parkinson Disease/physiopathology" [Mesh] AND "REM Sleep Behavior Disorder/physiopathology" [Mesh]	81
"Parkinson Disease/etiology" [Mesh] AND "REM Sleep Behavior Disorder/complications" [Mesh]	44
"Parkinson Disease/metabolism" [Mesh] AND "REM Sleep Behavior Disorder/metabolism" [Mesh]	28
"Parkinson Disease/physiopathology" [Mesh] AND "REM Sleep Behavior Disorder/complications"[ Mesh]	15

The articles that were included in our review were published within the past five years (2016-21), related to human subjects only without any restriction in age, gender, or demography. We selected the papers that were published in the English language only. All full-text articles were included without restrictions on the study type such as observational studies, clinical trials, cross-sectional studies, randomized control trials, systematic reviews, and traditional reviews. Any gray literature, animal studies, duplicate studies, articles that were published in a non-English language, and the papers published before the year 2016 were excluded from our review. 

Results

Search Outcome

Out of the total 1849 articles found in our initial search, 50 duplicate articles were removed which left us with 1799 articles. Thereafter, we applied our inclusion/exclusion criteria as mentioned and we were able to obtain 196 peer-reviewed, full-text articles in the English language. We screened these articles by their title and after a thorough study of the abstracts and the full text, selected 22 studies for quality appraisal.

**Figure 1:**
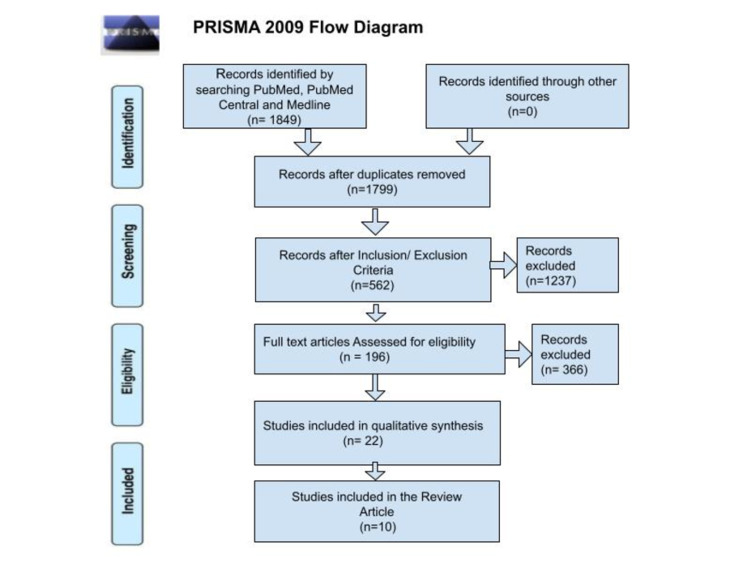
The PRISMA flow diagram Adapted from Moher D et al. [12] PRISMA: Preferred reporting items for systematic reviews and meta-analysis

Two authors meticulously assessed the selected studies for qualitative synthesis using different quality assessment tools. We used the Newcastle-Ottawa Scale (NOS) for observational studies, the PRISMA checklist for systematic reviews, the scale for the assessment of narrative review articles (SANRA) checklist for literature review articles, and the Cochrane risk-of-bias tool for randomized controlled trials. Ten studies were finalized to be included in our review as they fulfilled 70% criteria in their respective assessment tools. The PRISMA Flow Diagram is shown in Figure 1 [12]. 

Discussion

We will be elaborating on the pathophysiology, clinical presentations, and management of PD and RBD in this section. We will be discussing the conversion of RBD into neurodegenerative diseases and summarizing the various studies showing the close link between PD and RBD.

Pathophysiology and Clinical Implications of PD and RBD

Parkinson’s Disease (PD):The extrapyramidal nerve tract of the midbrain controls voluntary movements, posture, gait, and autonomic activities. The destruction of the dopaminergic neurons in the extrapyramidal nerve tract and the deposition of α-synuclein proteins, known as Lewy bodies, in the central, autonomic, and peripheral nervous systems lead to PD [13]. The major neurotransmitter involved in the pathogenesis of PD is dopamine but other neurotransmitters such as acetylcholine, gamma-Aminobutyric acid (GABA), and glutamate imbalance also contribute to PD. After the loss of dopaminergic neurons of up to 80% in the basal ganglia and substantia nigra pars compacta, patients with PD start developing motor manifestations [14]. There are six stages of neuropathological changes that occur in PD. The first two stages are the presymptomatic stages with the neurodegeneration limited to the medulla oblongata and the olfactory bulb. In the next two stages, patients start to develop symptoms with the involvement of substantia nigra and other nuclei of the midbrain and forebrain. The neocortex is affected in the last two stages and is associated with severe clinical presentations [4]. 

**Figure 2:**
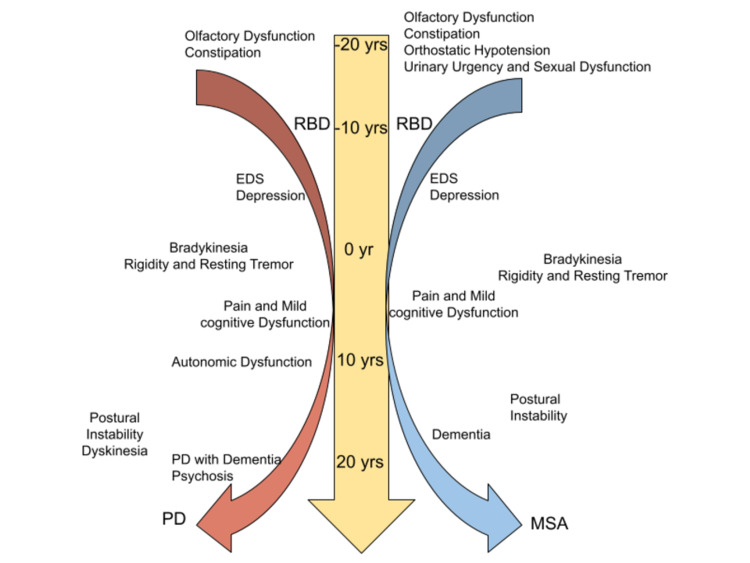
The timeline of RBD and other non-motor manifestations during the development of parkinsonism Adapted from Hong Jin et al. [10] EDS: Excessive daytime sleepiness; MSA: Multiple system atrophy; PD: Parkinson’s disease; RBD: Rapid eye movement sleep behavior disorder

The clinical manifestation of PD can be motor and non-motor symptoms. Patients present with bradykinesia (slowness of initiation of voluntary movements), muscular rigidity, resting tremor, pill-rolling tremor (circular movement of the thumb and index finger), postural instability, hypomimia (expressionless face), micrographia (small handwriting), shuffling gait, loss of balance, and falls [4, 15, 16]. The non-motor symptoms include autonomic dysfunction, sleep disturbances, and cognitive and psychiatric disturbances. Patients may present with anhedonia, loss of smell and taste, mood disturbances, excessive sweating, fatigue, constipation, postprandial fullness, RBD, nightmares, EDS, and anxiety [4, 16]. These symptoms have been reported to be present as early as 10 or more years before the initiation of motor symptoms in PD and become more severe with advancing disease [4]. These non-motor symptoms are also considered to be the prodromal biomarker for the early detection of PD [14]. Figure 2 demonstrates the timeline of the development of non-motor manifestations of parkinsonism. 

About 75-80% of patients with PD are at risk of developing sleep-wake disturbances over the course of the disease [17]. Disruption in the circadian rhythm, RBD, insomnia, EDS, vivid dreaming, and sleep apnoea or sleep-disordered breathing are the most common disorders seen in patients with PD [18-20]. The sleep disorders in PD have a severe impact on the quality of life of patients and marks a decline in cognitive performance and productivity, as well as major social and economic impacts [17, 18, 19, 21]. The exact underlying pathophysiology of sleep-wake disturbances in PD is still not clear. However, it is said to be caused by many factors leading to changes in the sleep-wake regulatory centers with impairment and imbalance of the neural circuits [17, 18]. They are often associated with the faster progression of motor symptoms in PD [22]. Therefore, early detection of sleep disorders is vital and it is done by polysomnography (PSG), which is the gold standard test for the detection of these disorders [23].

REM behavior disorder (RBD): Michel Jouvet was the first person to explain the brain circuitry of REM sleep. His investigation led to the identification of the dorsal pontine brainstem as the source of muscle atonia during REM sleep. Several decades of experiments have confirmed the region of the pons known as sublaterodorsal nucleus (SLD) - predominantly glutaminergic - as the location for the generation of atonia. Along with SLD, the pre-locus coeruleus region and the caudal laterodorsal tegmental nucleus (cLDT) are the components for the executive pontine circuit element in REM sleep. The GABAergic or glycinergic neurons of the ventral medulla (vM) and the glycinergic interneurons of the spinal ventral horn goes through synaptic activation, which is mediated by the glutamatergic neurons of the ventral SLD. This leads to motor atonia and temporary paralysis of the skeletal muscles during REM sleep [24-26]. 

Although the exact pathophysiology of RBD is still unclear, several neuroimaging studies in animals and humans have shown that the degeneration and damage in the excitation/inhibition of the brain circuitry of REM sleep including the SLD and vM lead to loss of atonia and dream enactment [24, 25]. Additionally, a neuromelanin MRI study done in humans showed that the RBD patients who had increased muscle tone had a decrease in intensity within the locus coeruleus/subcoerulus, and other imaging studies have shown multiple changes in the neurotransmitter systems, including the cholinergic, noradrenergic, and dopaminergic circuits [5, 25]. We can classify RBD as iRBD and secondary to the use of medications such as antidepressants like selective serotonin reuptake inhibitors (SSRIs) and focal brain lesions such as tumors, strokes, and multiple sclerosis causing disruption of the structures of the pons involved in REM atonia [25-27]. 

A male-predominant disorder, RBD usually onsets in the fifth or sixth decade with a prevalence rate estimated to be in the range of 0.5-2%. It is fivefold more commonly seen in patients taking antidepressants and tenfold more common in patients with underlying psychiatric conditions [26]. Patients often present with a history of abnormal vocalization such as shouting, swearing, laughing, and crying; abnormal motor behaviors such as punching, kicking, biting, falling from the bed, or even simple limb jerks; vivid dreams typically with violent and unpleasant themes such as chasing or attacking by animals or humans and often act out the contents from their dream; and even possess a serious risk to their bed partners leading to sleep disturbances and serious injuries [25, 26, 28]. The frequency, range, and severity of motor symptoms are widely fluctuant in different individuals and may present with completely different symptoms every other night [25]. As patients are unable to walk, run or leave the room due to loss of postural control during REM sleep, they often fall out of the bed during dream enactment [28]. 

The diagnosis of RBD is often missed in regular clinical practice [29], therefore a screening questionnaire and comprehensive history from the patient and collateral information from their bed partners regarding the history of sleep-related behaviors is crucial for the assessment of RBD [25]. Apart from the clinical history, subsequent overnight PSG with multiple-limb electromyography and synchronized digital video monitoring is the gold standard test for RBD according to ICSD-3 (International Classification of Sleep Disorder) [25, 27]. The PSG should demonstrate the complex vocal or motor behaviors during REM sleep along with REM sleep without atonia (RWSA). Furthermore, the sleep behavior disorder should not be better explained by other sleep or mental disorders and medications for the definite diagnosis of RBD [25, 26]. The management of RBD includes behavioral and pharmacological treatment. The behavioral management includes assessment of the sleep environment and creating a safe environment by removing potential hazards such as sharp objects, placing soft mattresses, cotton padding, or pillows on the floor along with recommendations for the patient and the bed partner to sleep separately. The pharmacological treatment includes medications such as melatonin and clonazepam [26, 28, 30].

The Correlation Between RBD and PD

The close relationship between alpha synucleinopathies and RBD has come to light with recent advancements in studies. A study has shown that 94 percent of the patients with RBD had deposition of alpha-synuclein in their brains. The patients who had a greater density of deposition of alpha-synuclein were at a higher risk of developing RBD symptoms [10]. The longitudinal cohort studies have proven that the development of neurodegenerative diseases such as PD, DLB, and MSA was undeniable in iRBD patients [26]. About 98% of PSG confirmed RBD patients and 94% with probable RBD only by history had autopsy proven Lewy type synucleinopathy in an autopsy study done in 172 subjects [31]. 

RBD is considered as one of the strongest clinical predictors in developing PD and has been seen to be associated with more severe motor and nonmotor symptoms in PD patients and a poorer prognosis [3, 32, 33]. A study has reported that 81% of iRBD elderly male patients eventually developed parkinsonian syndrome and the mean duration between the diagnosis of RBD and the motor symptoms of PD has been reported to be about a decade [10]. The presence of RBD was more common in the rigid-akinetic subtype of PD than in the tremor dominant subtype and usually presented with hypomimia, hypophonia, and reduced arm swing [32, 33]. The non-motor symptoms include constipation, olfactory dysfunction, EDS, cognitive impairment, and even psychiatric symptoms such as mood disorders, hallucinations, and anxiety [34]. An observational study conducted on 162 subjects concluded that there is an increased risk of cognitive decline and dementia in presence of RBD in PD patients [35]. The deposition of Lewy body and neurite along with neural loss and gliosis in the brainstem areas that mediate REM sleep were found in the post-mortem study of brains of patients of RBD with comorbid PD [10]. A cohort study conducted using the open-access Parkinson’s Progression Markers Initiative (PPMI) database concluded that the presence of RBD in PD was associated with smaller volumes of the putamen, thalamus, and hippocampus [34]. In addition, changes in the left and right cingulum and left inferior occipital fasciculus have also been considered the potential landmarks in analyzing the brain changes that correlate RBD with PD [33]. 

The underlying alpha-synucleinopathy can be tested in RBD patients by identifying misfolded, pathological, and phosphorylated α-synuclein protein in peripheral tissues such as dermal nerve fibers and colon; and fluid samples such as salivary glands, parotid glands, submandibular glands, and cerebrospinal fluid) [25, 36]. Other less invasive bioimaging tests such as MRI and PET scanning may also be used to identify the dysfunctions correlating with α-synucleinopathies [25]. The evaluation of presynaptic nigrostriatal function using the dopamine transporter neuroimaging (DaTScan) can also be done which may show a decrease in the striatal tracer uptake [37]. However, all RBD may not be detected through DaTScan as the pathology of RBD precedes the gross dopaminergic changes seen in PD [25]. Additionally, EEG slowing during wakefulness may also be observed in more severe neurodegeneration [3]. These diagnostic tests are always not valuable and feasible in the clinical scenario. Therefore, a comprehensive clinical assessment would always be a better option in evaluating RBD and contemplating the risk of development of PD and other neurodegenerative diseases. There are no specific clinical therapies for α-synucleinopathies but, studies are going on about the neuroprotective compounds which may prevent these conditions. Early recognition and diagnosis, proper patient counseling, behavioral and pharmacological treatment of RBD are important to slow the progression of α-synucleinopathies [25]. 

Table 3 summarizes the various studies done to describe the correlation between RBD and PD.

**Table 3:** Table showing previous studies on the relationship between Parkinson’s disease and rapid eye movement behavior disorder. PD: Parkinson’s disease; RBD: Rapid eye movement sleep behavior disorder; RBE: Rapid eye movement sleep behavioral events; iRBD: Idiopathic rapid eye movement behavior disorder; PPMI: Parkinson’s progression markers initiative

Author	Year of Publication	Type of study	Purpose of the study	Result/ Conclusion
Friederike Sixel-Döring et al. [7]	2016	Observational study	To study about RBD and RBE not yet fulfilling diagnostic criteria for RBD as markers for neurodegeneration	In a two-year follow-up cohort study, RBD was found to be a robust marker of early PD and RBE should be considered as ‘RBD-prodromal’ state, which can also evolute into PD.
Hong Jin et al. [10]	2017	Review	To describe the clinical and pathological significance of RBD focusing on the nonmotor symptoms of PD	The study helps understand the strong link of iRBD with non-motor symptoms of RBD and also stresses that more studies are required for the analysis of RBD in PD.
Natalia Jozwiak et al. [35]	2017	Observational study	To study the existence of cognitive impairment in PD and RBD	The study concluded that wide neurodegeneration and increased cognitive decline in PD were associated with RBD patients.
Chahine et al. [3]	2017	Review	To provide an overview of sleep and wakefulness in PD	The study gives an idea about the impact of sleep-related dysfunctions such as RBD, circadian rhythm disorders in PD patients.
Sanne Kamps et al. [34]	2018	Observational study	To identify the biomarkers of PD progression	The multicenter cohort study with the use of the PPMI database concluded that the presence of RBD in PD patients showed smaller volumes of putamen, thalamus, and hippocampus.
David R. Shpreche et al. [31]	2018	Research article	To study the diagnosis of RBD for the prediction of alpha-synucleinopathies	RBD can be considered as the biomarker for the differential diagnosis of neurodegenerative diseases.
Min Li et al. [32]	2018	Review	To discuss the clinical assessment, biomarkers, and treatment of RBD and its relationship with neurodegenerative diseases	Further research and treatment trials are required to determine the effectiveness of the intervention of RBD to slow down the process of neurodegeneration.
Barone and Henchcliffe [33]	2018	Review	To study the link between RBD and alpha-synucleinopathies	Improved recognition of RBD leads to early diagnosis of alpha-synucleinopathies
Sanne K. Meles [38]	2018	Observational study	To understand the metabolic pattern of iRBD and its association with early PD	The results showed that iRBD is an early manifestation of PD.
Zhemin Huang et al. [37]	2019	Observational study	To understand the correlations between dopaminergic dysfunction and abnormal metabolic network activity in REM sleep behavior disorder	The dysfunction in many neurotransmitters along with the nigrostriatal dopaminergic dysfunction is related to iRBD in PD.

Limitations

There are several limitations to this review because the articles included in this review were human studies written in the English language only and were published within the past five years. It might have ruled out many papers with information relevant to this topic and many animal studies done regarding the pathology of RBD might have been missed in this review. Furthermore, the studies in this paper had smaller study groups. So, it cannot be generalized and the relation between RBD and PD still requires a lot of further longitudinal studies and clinical trials to better understand the underlying pathophysiology.

## Conclusions

To summarize, apart from the motor symptoms in PD, increasing advancements in studies have considered RBD and other non-motor symptoms as the strongest clinical predictors of PD. The phenoconversion of RBD into PD takes about a decade and seems to be associated with more severe motor and non-motor symptoms with a poorer prognosis. The high prevalence of RBD in PD patients has shown a significant reduction in the quality of life and even early institutionalization. With this review, we would like to highlight the significance of recognition and diagnosis of RBD for the early detection of PD by clinicians. Further clinical research, longitudinal studies, and treatment trials are required to determine the effectiveness of the intervention of RBD as a therapeutic window to slow down the process of neurodegeneration.
